# Real-time immunophenotypic shifts in pediatric B lymphoblastic leukemia providing implications for minimal residual disease detection

**DOI:** 10.1515/almed-2024-0202

**Published:** 2025-05-05

**Authors:** Omer Javed, Neelum Mansoor, Naeem Jabbar, Hamza Khan, Talha Israr, Sidra Maqsood, Saba Jamal, Fatima Meraj

**Affiliations:** Department of Hematology, 586034 Indus Hospital and Health Network, Karachi, Pakistan; Department of Cytogenetics and Hematology, Indus Hospital and Health Network, Karachi, Pakistan; Department of Pediatric Oncology, Indus Hospital and Health Network, Karachi, Pakistan; ORIC, Indus Hospital and Health Network, Karachi, Pakistan; Clinical Laboratories and Blood Transfusion Services, Indus Hospital and Health Network, Karachi, Pakistan

**Keywords:** acute lymphoblastic leukemia, blast cells, flow cytometry, immunophenotypic modulation, minimal residual disease

## Abstract

**Objectives:**

Disease monitoring in acute leukemia management is crucial for risk stratification and chemotherapy response assessment. Minimal residual disease (MRD) testing is the most reliable tool, measuring antigenic expression differences between leukemic cells, hematogones, and mature benign B-cells. Modulation of antigen expression during treatment under chemotherapeutic drugs may complicate MRD analysis and detection. The present study investigates immunophenotypic modulation (IM) during chemotherapy phases in children with acute lymphoblastic leukemia (B-ALL), using a modified BFM protocol, and its potential implications for MRD detection.

**Methods:**

The study was conducted at the Hematology Department, Indus Hospital and Health Network, Karachi. All MRD positive cases of pediatric B-ALL (1 month −16 years of age) were included, from April 2019- March 2022. MRD was done on bone marrow aspirate using 8-color flow cytometry. The data from 203 patients, who were MRD positive in bone marrow throughout the evaluation period was considered. The IM was assessed by comparative analysis of the changes in mean fluorescence intensity (MFI) of nine highly relevant antigens expressed by the leukemic cells.

**Results:**

Statistically significant changes in the MFI levels of antigens were observed in leukemic blasts and mature benign B-cells. All the analyzed samples revealed IM to different extents. Our results confirm the presence of immunophenotypic changes for CD10, CD19, CD34, CD45, TdT, and CD66 during chemotherapy.

**Conclusions:**

Measuring the MRD assists in monitoring the disease, however, IM of the different antigens expressed by the leukemic blasts should be considered and cautiously analyzed to prevent erroneous results.

## Introduction

Acute lymphoblastic leukemia (ALL) is the most common childhood cancer, with 80–85 % of cases being B-cell acute lymphoblastic leukemia (B-ALL) [1]. Flow cytometry is commonly used to study the immunophenotype of the malignant cells [2]. Disease monitoring in the management of acute leukemia is crucially important in terms of risk stratification and response to chemotherapy for which minimal residual disease (MRD) testing is the most reliable strategy, evolved over the last few years [2]. MRD testing by flow cytometry or polymerase chain reaction (PCR) is an important prognostic indicator and is pivotal in predicting outcomes [3]. Measurement of MRD by flow cytometry is based on the principle that leukemic cells present unusual antigenic patterns that distinguish them from maturing precursor cells i.e. hematogones [4]. It is a widely used modality to assess disease response to therapy with a very high sensitivity.

Flowcytometric detection of residual blasts, hematogones and variation in the expression of different antigens under the influence of chemotherapy and during the course of disease has gained a lot of research interest worldwide. It is analyzed at various time points during treatment to monitor remission status [5]. Antigenic variation occurs on residual blasts of B-ALL patients during treatment, particularly during the induction chemotherapy which may cause difficulty in analysis and interpretation of MRD [6].

Different markers used in diagnostic phenotype and MRD monitoring show modulation due to chemotherapeutic agents. Literature has shown CD10, CD20, CD34, TdT and CD45 expression modulation, particularly during the glucocorticoid phase (GC phase) of induction therapy [7]. For MRD assessment, such phenotypic shifts pose a particular challenge since they can cause misinterpretations in terms of false negative or false positive reporting of results.

A recently published study showed a relatively higher frequency of MRD in our pediatric B-ALL patients however, to our knowledge, very little is known about immunophenotypic modulation patterns of acute leukemia in our settings [8]. This study extensively demonstrates the expression and variation of immature and lineage-specific markers. Through a single-center study, a good sample size would help to compare our data with other studies and identify significant antigenic shifts that will provide insight into MRD interpretation. We aimed to study the trends in immunophenotypic modulation of leukemic cells and analyze patterns in comparison to non-leukemic B-cells within the same sample which may coexist during different phases of chemotherapy.

## Materials and methods

### Patients

It is a retrospective and observational study conducted at the Hematology Department of Indus Hospital and Health Network, Karachi, Pakistan from April 2019 to March 2022. All newly diagnosed patients with B-ALL, aged 1 month to 16 years were included. As per National Cancer Institute (NCI) risk stratification criteria all patients received vincristine, asparaginase, dexamethasone, doxorubicin/daunorubicin, 6 MP, and intrathecal methotrexate using modified Berlin-Frankfurt-Munster (BFM) chemotherapy protocol. This protocol comprises of one-week prednisone prophase with 60 mg/m^2^/day per oral or intravenous followed by determination of prophase response on day 8 using peripheral blood blast percentage.

### Flow cytometry

Flow cytometry was used for the diagnosis and MRD detection. The immunophenotypic modulation (IM) in antigen expression of TdT, CD34, CD10, CD19, CD20, CD45, CD13, CD33, and CD66 was observed. Antigen expression was analyzed by mean fluorescence intensity (MFI) values of leukemic and non-leukemic mature benign B-cells.

Flow cytometry analysis was conducted utilizing a FACS Canto II flow cytometer (Becton Dickinson, Franklin Lakes, NJ) with FACS Diva software for instrument configuration and sample acquisition throughout this study. Event acquisition for the two-tube, eight-color panel incorporated all mononuclear events (distinguished by low log SSC vs. CD19) with stop counts set at>500,000 events. A minimum number of 500,000 events were acquired for all specimens. Data analysis was executed on list mode files utilizing FACS DIVA software version 8.0.2. A unified gating approach was applied across the samples by two independent observers following the same protocol. The analysis gating strategy for the eight-color panel involved the inclusion of all B-cells via a CD19/log SSC gate, followed by further characterization of immature B-cells based on the expression of other antibodies in the panel. The uniform gating strategy utilized in the eight-color assay incorporated parent gates isolating mononuclear cells and singlets followed by a logical gate initially based on positive CD19/log SSC results, followed by additional gating for B lymphoblasts and hematogones utilizing CD19 by CD45 and CD19 by CD10. Minimal residual disease (MRD) positivity was determined by the presence of an abnormal blast population comprising at least 0.01 % of total mononuclear cells.

Isolation of benign mature B-cell population is done based on low SSC properties, bright CD45, negative CD10, and bright CD20 expression in CD19 gated cells.

### MRD at different phases of chemotherapy

MRD results were collected and compared from the time of diagnosis day 0, at day 35 after induction chemotherapy, at day 52 after consolidation chemotherapy, and during maintenance chemotherapy at day 78. For the analysis of the immunophenotypic shift/modulation on blasts, only patients who persisted with MRD positivity through all phases were selected and included in the study.

Diagnostic flow cytometry was performed in peripheral blood or bone marrow aspirate whereas MRD was exclusively done on bone marrow aspirate samples.

### Statistical analysis

SPSS version 24.0 was used to calculate the frequency of gender, median and interquartile ranges (IQR) for the age and different antigens CD10, CD45, CD20, TdT, CD34, CD19, CD66, CD13 and CD33. The Wilcoxon signed-rank test is used to compare the median of MFI values at diagnosis and subsequent time points of MRD. The Mann–Whitney U test was applied to determine significant differences in the expression of antigens. A p-value of <0.05 was considered significant.

## Results

A total of 203 cases of B-ALL were included that persisted with MRD positivity throughout the treatment course. The group had a median age of 5.7 years (IQR 3.0–8.0). In this cohort, 123 (60.6 %) were males and 80 (39.4 %) were females. We studied the expression of nine antigens in our B-ALL MRD panel of leukemic blast cell population CD10, CD45, CD20, TdT, CD34, CD19, CD66, CD13, CD33, and three antigens in benign (mature benign B lymphocytes) cell population CD45, CD19, and CD20.

**Figure 1:**
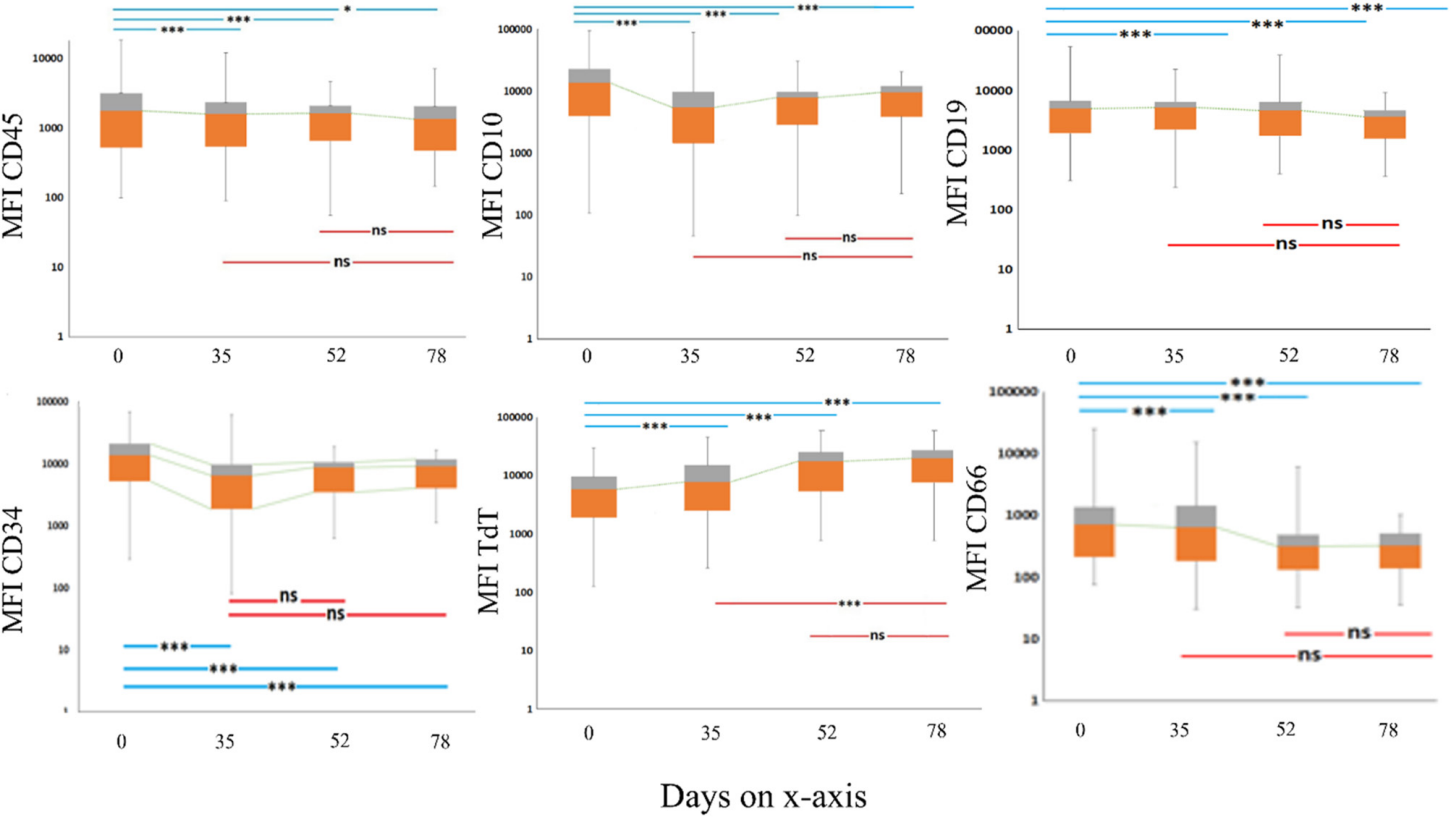
Modulation of leukemic cells. Changes in the MFI of CD10, CD45, CD34, CD66, CD19, and TdT during chemotherapy. D0, D35, D52 and D78. The levels of statistical significance are given in each box above the respective interval investigated (ns=not significant; *= p<0.05; ***= p*<*0.01).

We observed statistically significant changes in the MFI values of six antigens expressed by the blasts: CD10, CD45, CD34, CD66, CD19, and TdT (Figure 1).

The box covers the interquartile interval (lower and upper quartiles), where 50 % of the data is found and whiskers (minimum-maximum) summarize the distribution of data. Median values of the cohorts are represented by dashed lines reflecting their evolution.

### Antigen modulation in blasts

CD10: There was a consistent and significant downmodulation observed during the interval day 0 to day 35 (Z: −7.039; p<0.001), day 0 to day 52 (p<0.001), and day 0 to day 78 (p<0.001). Conversely, between the interval day 35–78 and day 52–78, a nonsignificant up modulation was noted.

CD45: Notably, significant and stable down modulation was observed on day 35 (p=0.003), day 52 (p<0.001), and day 78 (Z: −2.181; p=0.029) in the bone marrow.

CD34: A significant down modulation was observed from day 0 through to the subsequent phases i.e. day 35, day 52 and day 78 (p<0.001), moreover between interval phases a non-significant variation in expression was observed within phases.

CD66: a significant and stable down modulation was seen on day 0–35 (p<0.001), day 0–52 (p<0.001) and day 0–78 (p<0.001). A non-significant downmodulation was observed day on day 35–78 and day 52–78.

CD19: significant stable downmodulation was seen on day 0–35 (p<0.001), day 0–52 (p<0.001), day 0–78 (Z: −4.024; p<0.001).

TdT: Significant stable up modulation was observed on day 0–35 (p<0.001), day 0–52 (p<0.001), day 0–78 (p<0.001) and day 35–78 (p=0.021).

CD20: Small but reversible upmodulation was detected in both interval of days 0–35 and 0–78 while downmodulation for 0–52. However, the change was found to be non-significant for all three time points. A significant up-modulation was observed between intervals of day 35–78 (p=0.005).

CD33: Our study showed a variable trend in CD33; a non-significant but stable up modulation in CD33 expression was observed from day 0–35, 0–52 and 0–78 while between interval phases a stable down modulation was seen between days 35–78, and 52–78.

CD13: Similar to CD33, the expression of CD13 showed variable trends between phases i.e. shows a non-significant reversible up modulation from 0 to 35 which was reversible in the following phases.

### Antigen modulation in mature benign B-cells

**Figure 2:**
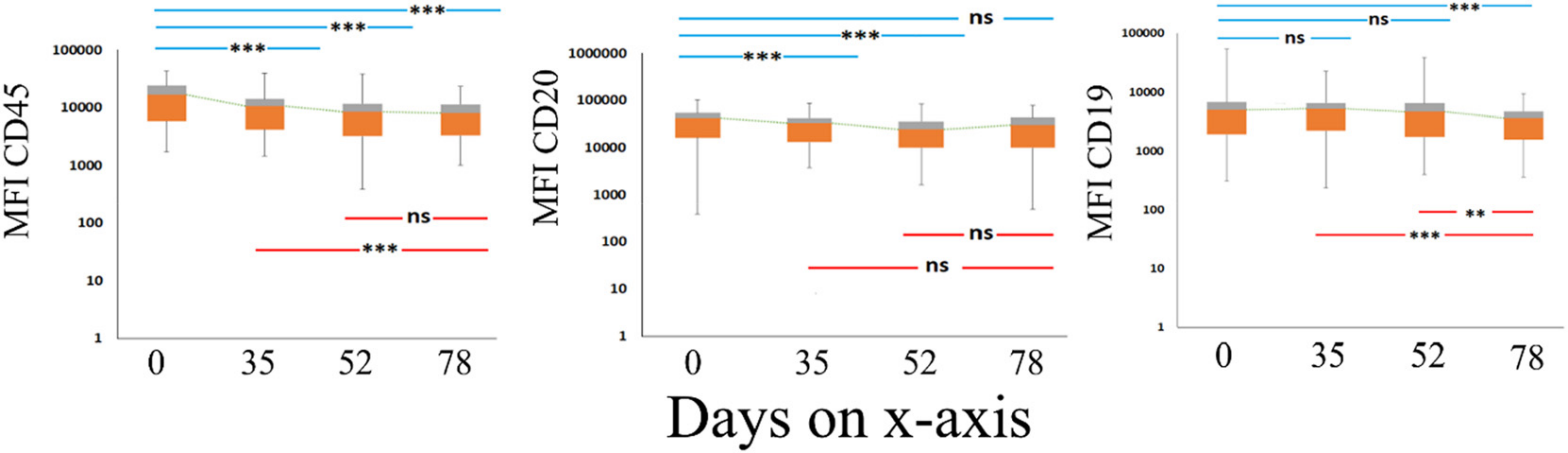
Modulation of benign residual B lymphocytes. Changes in the MFI of CD45, CD20, and CD19 during chemotherapy. D0, D35, D52and D78. The levels of statistical significance are given in each box above the respective interval investigated (ns=not significant; *= p<0.05; ***= p*<*0.01).

Similarly, to observe the variation of antigenic expression and the effect of therapy on antigenic expression in mature benign B lymphocytes within the sample, we examined trends of CD45, CD20, and CD19 expression between day 0, day 35, day 52, and day 78, respectively. In mature benign B lymphocyte population (Figure 2), CD45 expressions show stable significant down-modulation on day 0–35 (p<0.001), day 0–52 (p<0.001), day 0–78 (p<0.001) and day 35–78 (p=0.003); A stable significant downmodulation was observed in CD20 expression between intervals days 0–35 (p<0.001) and day 0–52 (p<0.001). For CD19 expression, a stable non-significant down-modulation was seen on day 0–35 and day 0–52; however, a significant down-modulation was seen on day 0–78 (p<0.001), day 35–78 (p=0.003) and day 52–78 (p=0.017).

The box covers the interquartile interval (lower and upper quartiles), where 50 % of the data is found and whiskers (minimum–maximum) summarize the distribution of data. Median values of the cohorts are represented by dashed lines reflecting their evolution.

### Comparison of mature benign B lymphocytes and leukemic cells

We have additionally compared the expressions of CD45, CD20, and CD19 in leukemic cells to benign cells, revealing a noteworthy difference at the time of diagnosis and consistently across various phases of chemotherapy, as illustrated in Table 1.

**Table 1:** Comparison of IM in residual B-lymphocyte population and leukemic blast cell population by Mann–Whitney test.

Median (IQR)	Day 0	p-Value	Day 35	p-Value	Day 52	p-Value	Day 78	p-Value
CD45–L	1,266 (532.8–2,700)	0.000^a^	1,060.8 (550.9–1,814.7)	0.000^a^	997.5 (661.3–1,466.9)	0.000^a^	889 (479.1–1,588.3)	0.000^a^
CD45-B	10,675 (5,964–17,906)		6,544 (4,239–9,842)		5,395 (3,213–8,409)		4,622 (3,340–8,041)	
CD20-L	1,071.6 (360–3,591.6)	0.000^a^	1,233.5 (506.2–2,781.5)	0.000^a^	935 (524.4–2,165)	0.000^a^	1,389.5 (705.5–3,260.7)	0.000^a^
CD20-B	26,091 (16,393–37,556)		20,112 (13,229–30,325)		14,696 (10,011–26,219.5)		20,085 (9,977–34322)	
CD19-L	2,376 (1,533–4,571)	0.007^a^	1,968 (1,206–3,309)	0.000^a^	1,474.2 (1,047.7–2,009.2)	0.000^a^	1,501.3 (1,037.3–2,231.5)	0.001^a^
CD19-B	3,079 (1,919–4,846)		3,012 (2,219–4,299)		2,890 (1,754–4,717)		2,142 (1,572–3,120)	

Significant p-value^a^, L, leukemic blast cell population; B, benign cell population (residual B-lymphocytes), IM, immunophenotypic modulation.

## Discussion

The prognostic significance of measuring MRD is firmly established in pediatric ALL. Post-chemotherapy, immunophenotypic alterations frequently arise in leukemic cells, primarily attributable to Glucocorticoid (GC) treatment. The present study reported statistically significant changes in the MFI values of six CD markers expressed by the leukemic blasts: CD10, CD45, CD34, CD66, CD19, and TdT; In contrast, the changes were non-significant for CD20, CD13, and CD33.

This is of concern because important markers such as CD34, CD10, CD19, CD45 can be modulated, which can impact the detection of MRD. The changes may involve the intensity of expression, loss or gain of antigens, or lineage change [9]. A study reported that there is a ∼25 % chance of missing MRD detection in primary markers [10]. Some other studies have also reported antigen modulation in leukemic cells after Berlin-Frankfurt-Munich induction protocol [6], [7], [11], which was in concordance with our study.

Stable downmodulation of CD10 was observed in our study till day 78 as comparable with those at diagnosis, Burnusuzov and colleagues also observed. [6]. The other study by Dworzak and colleagues reported significantly declined CD10 expression until day 35 and recovered thereafter till day 78 to values comparable with those at diagnosis [7]. There was a significant down-modulation in expression of CD34 in our cohort which was, although reversible and showed trends of re-increase over the subsequent phases, but not to the limit of the diagnostic levels. Similar to ours, Dworzak et al. observed a reversible modulation particularly in CD34 expression which was greatly variable during follow-up. Furthermore, there was a tendency for a re-increase in the post-induction phase in certain patients following the tapering of glucocorticoids. This reversible pattern could be attributed to the glucocorticoid-containing chemotherapy, aligning with the schedule of glucocorticoid therapy [12].

Certain other studies also reported differences in the documented expression of CD34 during chemotherapy. Gaipa and coworkers [13] reported a down modulation of CD34, contrasting with the findings of Thulasi et al. [14] and Cáp and coworkers [15], who observed an increase in CD34 expression following the tapering of GCs. Our study contributes to the literature by revealing a significant reversible antigenic modulation in CD34 expression, further contributing to the understanding of its behavior during chemotherapy.

In contrast to Dworzek and colleagues, our study revealed a significant and stable down-modulation in CD45 expression throughout the time interval from day 0 to 78 [7]. Additionally, Gaipa and coworkers reported no significant change in CD45 expression at all observed time points [4] The stable expression of CD66 was observed in our study with significant downmodulation throughout the different phases of chemotherapy. The study by Ismail M et al. also reported the down-regulation of CD66 [16].

Literature shows an initial CD19 up-modulation in the initial weeks of chemotherapy till day 15, followed by a noteworthy down-modulation in subsequent phases, this aligns with the findings in our study. We observed a stable and significant down-modulation from day 0 to 78, with a non-significant down modulation noted on days 35–78. Consistently, Chowdhury et al. also reported a down-modulation of CD19 after one week of chemotherapy [17]. In our study, significant and stable up-modulation was observed in TdT expression (after prednisone) which is in discordance with a study by Akiyama and coworkers that reported down-modulation of TdT gene expression on B-ALL by prednisone [18].

We have reported the reversible modulation of CD20 marker which was not statistically significant. Burnusuzov et al. reported a small non-significant up-modulation of CD20 [6]. The other study reported upregulation of CD20 marker [8]. Expression and persistence of documented myeloid antigens CD13 and CD33 was highly variable.

Reversible up-modulation was noted in CD13 expression. Among the 168 samples positive for CD13 at day 0, reproducible results were observed in 17 cases (10 %) that remained positive at day 35, and 7 cases (4 %) that were positive at day 52. In the case of CD33, a stable up-modulation was observed from day 0 to 78. Again, out of the total 173 cases positive for CD33 at day 0, reproducible positivity and persistence of positive expression were observed in 10 cases (5.7 %) at day 35, 8 cases (4.6 %) at day 52, and 5 cases (2.8 %) at day 78.

In our investigation, we explored the targeted impact of IM exclusively on the leukemic population. This inquiry involved an analysis of variations in the expression of CD19, CD20, and CD45 within the mature benign B-lymphocytes. Previous studies have highlighted the regulated nature of antigen expression patterns in normal mature B-cells (lymphocytes). Notably, a study demonstrated statistically significant downmodulation of CD19 between day 15 and day 33 (p=0.008) in benign cell CD19 expression [11]. However, our findings revealed a non-significant, yet stable, downmodulation from day 0 to day 35.

Furthermore, published results indicate a higher level of up-modulation of CD20 and CD45 expressed by B-lymphocytes compared to that of leukemic cells [11]. In our study, we reported reversible modulation in leukemic cells and stable down-modulation of CD20 in benign cells. Additionally, our findings indicated stable down-modulation for CD45 in both leukemic and benign cell populations. This nuanced understanding of antigen expression patterns contributes to a more comprehensive assessment of the immunomodulatory effects on both normal and leukemic B-cell populations.

During the initial induction therapy, it becomes quite challenging to differentiate and detect diseased blasts, implying the immunophenotypic alterations under the effect of chemotherapeutic agents, presence of hematogones and in absence of other leukemia associated immunophenotype (LAIP). Employing eight-color flow cytometry and comprehensive antibodies, along with detailed knowledge and trends in mind of the immunophenotypic shifts under the effect of therapy can still be predicted and studied in the context of labelling the disease relying on the modulation of various immune phenotypes of malignant and normal B-lymphocytes. With the use of other stable CD markers in the MRD panel, it becomes feasible to unequivocally differentiate the MRD population in all samples.

Performing gating in flow cytometry to measure MRD in acute lymphoblastic leukemia has the potential for subjectivity and variability in the gating process. Different operators may define gates differently, which can lead to inconsistencies in the results. The presence of overlapping populations and low levels of residual disease can make it challenging to accurately identify and quantify the target cells. However, in our study, the gating strategy was unified across the samples as all operators strictly follow the same protocol of gating that is clearly defined in standard operating procedures, and each case is extensively analyzed by two observers independently. Therefore, the variability in analysis and interpretation is markedly minimized in this study.

## Limitations

The study’s limitation lies in its exclusive focus on antigenic modulation in pediatric ALL cases, without extending the analysis to adult ALL cases. We strongly advocate for exploring diverse marker combinations, including novel ones, to enhance the assay’s sensitivity and specificity.

## Conclusions

In conclusion, this study provides compelling evidence of significant immunophenotypic modulation in pediatric B-ALL patients undergoing chemotherapy. The observed fluctuations in MFI across key antigens like CD10, CD19, CD34, CD45, TdT, and CD66 underscore the dynamic nature of leukemic blasts under treatment. While MRD testing remains a cornerstone of disease management, the potential for chemotherapy-induced antigen modulation necessitates a cautious approach to MRD analysis. Ignoring these changes risks generating inaccurate MRD assessments, which could ultimately impact patient risk stratification and treatment strategies. Therefore, meticulous analysis and consideration of immunophenotypic shifts are crucial for optimizing the reliability of MRD detection. Future research should focus on further elucidating the mechanisms driving these modulations and developing standardized protocols to mitigate their impact on MRD interpretation.
